# Impact of frailty on rehabilitation exercise adherence in patients with ischemic stroke

**DOI:** 10.3389/fmed.2025.1679267

**Published:** 2025-11-07

**Authors:** Qingwen Long, Li Wu, Yongli Li, Yi Wu

**Affiliations:** 1 Department of Neurology, Affiliated Hospital of North Sichuan Medical College, Nanchong, China; 2 Department of Nursing, North Sichuan Medical College, Nanchong, China; 3 Department of Gastrointestinal Surgery II, Affiliated Hospital of North Sichuan Medical College, Nanchong, China; 4 Guang'an District People's Hospital of Guang'an City, Guang'an, China; 5 Institute of Hepatobiliary, Pancreatic, and Intestinal Diseases, North Sichuan Medical College, Nanchong, China

**Keywords:** frailty, rehabilitation exercise, adherence, ischemic stroke, threshold effect analysis

## Abstract

**Objectives:**

To identify the threshold effect of frailty on rehabilitation exercise adherence in patients with ischemic stroke.

**Methods:**

The study included 307 patients diagnosed with ischemic stroke who were given a questionnaire comprising a general information form, the Rehabilitation Adherence Assessment Scale, and the Frailty Assessment Scale. Univariate and multivariable linear regression were employed to determine factors influencing rehabilitation exercise adherence. Subsequently, restricted cubic splines were utilized to fit a smooth curve and detect potential threshold effects.

**Results:**

The average score for the rehabilitation exercise adherence index was (54.83 ± 9.32), while the average frailty score was (4.59 ± 2.14). Through univariate analysis, it was found that gender, marital status, living arrangement, household registration type, number of medications, and level of independence are factors influencing rehabilitation exercise adherence. Restricted cubic spline analysis revealed a non-linear relationship between frailty and rehabilitation exercise adherence. The association between frailty score and rehabilitation exercise adherence was found to be insignificant when the score was ≤3.98. Conversely, exceeding this threshold revealed a significant decline in the adherence index, with each additional frailty point correlating with a 2.56-point decrease (*p* < 0.001).

**Conclusion:**

Rehabilitation exercise adherence is moderate among patients with ischemic stroke, while the prevalence of frailty is notably high. A frailty score above 3.98 might serve as an early indicator of its impact on adherence. Accordingly, rehabilitation programs need to be adjusted to accommodate disease characteristics and sociodemographic factors.

## Introduction

Ischemic stroke, a prevalent form of acute cerebrovascular injury, accounts for approximately 80% of all cerebrovascular disorders (1). It results from cerebral arterial narrowing or occlusion, leading to cerebral tissue necrosis and insufficient cerebral perfusion, and constitutes a major cause of disability and mortality (2, 3). The global burden of disease data show a steady increase in the incidence, disability, and mortality associated with ischemic stroke (4). Approximately 34% of worldwide healthcare expenditure is currently allocated to treatment and care, representing a considerable burden on public health systems (5, 6). Amid rapid population aging and accelerating urbanization, China’s stroke burden is exploding; the nation accounts for one-third of global incidence and mortality, incurring profound medical and societal costs (7).

Approximately 80% of ischemic stroke survivors are left with varying degrees of functional impairment, further compounding the disease burden. According to evidence-based medicine, rehabilitation exercise is recognized as the most effective intervention for reducing disability and enhancing activities of daily living (8, 9). It facilitates neural reorganization and recruits residual neurons to compensate for damaged tissue, thereby maximizing functional recovery (10, 11). Rehabilitation exercise adherence denotes the extent to which patients comply with professional guidance and engage in prescribed training activities; high adherence is pivotal for optimizing recovery and clinical outcome (12). Stroke recovery is inherently prolonged, and rehabilitation exercise necessitates a gradual and sustained endeavor. Surveys suggest that approximately 50% of patients fail to sustain adequate adherence, significantly diminishing therapeutic effectiveness (12–14).

Post-stroke frailty, a common clinical syndrome in ischemic stroke survivors, is defined by dysregulation in various physiological systems and increased vulnerability, resulting in compromised homeostatic function (15, 16). The occurrence of frailty in stroke patients with poor baseline status undergoing prolonged inpatient rehabilitation is reported at 62.9% (17). Emerging evidence indicates that frailty not only directly impairs rehabilitation exercise adherence but also heightens the risk of adverse outcomes (18–20). Research on the relationship between rehabilitation exercise adherence and frailty is currently limited to broad associations and general patterns, with a notable absence of threshold-effect analyses. Therefore, on the basis of identifying influencing factors, this study further examined the curvilinear relationship and threshold effect between frailty and rehabilitation exercise adherence, providing an empirical basis for clinical intervention and evaluation.

## Methods

### Study design

We conducted a cross-sectional survey among patients with ischemic stroke. The study was approved by the relevant institutional ethics committee, and informed consent was obtained from all participants. This report adheres to the STROBE Statement guidelines.

### Participants

A convenience sample was recruited among stroke patients attending two tertiary hospitals in Sichuan Province between January and May 2025. Inclusion criteria: (1) Ischemic stroke confirmed by CT or MRI and diagnosed in accordance with the 2019 Chinese Guidelines for Major Cerebrovascular Diseases (21); (2) Age ≥ 45 years; (3) Clinical stability following treatment; (4) Hospitalization for ≥ 14 days; (5) Limb dysfunction (muscle strength ≤ grade 4); (6) Participation in rehabilitation exercise for ≥ 1 week; (7) Consent obtained after informing the participants. Exclusion criteria: (1) Concomitant hemorrhagic stroke; (2) Cardiac or pulmonary insufficiency, hepatic or renal failure, or malignant tumor; (3) Impaired consciousness, cognition, mental status, or language function. According to the literature (22), the required sample size is typically 10–15 observations per variable. With 20 variables in this study and accounting for a 20% rate of missing or invalid questionnaires, the calculated minimum sample size is 240 participants. Data was collected the day before discharge. Out of 320 questionnaires distributed, 307 were valid after excluding incomplete or inconsistent responses, resulting in an effective response rate of 95.9%.

### Assessments

#### General demographic and clinical characteristics form

Investigators developed the form by conducting a literature review, which encompasses patients’ sociodemographic and clinical data. Variables include sex, age, education level, living arrangement, marital status, occupation, household registration type, medical insurance, smoking and alcohol status, number of medications, number of strokes, comorbidities, and level of independence. The number of medications was defined by the number of chronic conditions for which the participant had been prescribed and regularly took the corresponding medication for ≥3 months. The level of independence was quantified using the Barthel Index. The scale comprises 10 items, each scored 0, 5, or 10 according to the degree of task completion, yielding a total score ranging from 0 to 100, with higher scores indicating greater independence in activities of daily living. Participants were categorized into three groups: completely dependent (≤40), minimally independent (41 ~ 60), and mostly independent (>60).

### The questionnaire on exercise adherence

The scale, developed by Lin et al. (23), comprises three dimensions: physical participation in exercise adherence (items 1–8), exercise outcomes monitoring adherence (items 9–11), and seeking timely advice during exercise adherence (items 12–14). It consists of 14 items rated on a 4-point scale (1–4), yielding a total score between 0 and 56, where higher scores indicate stronger adherence.

The scale exhibited excellent internal consistency in this study, as indicated by a Cronbach’s *α* value of 0.869. The study quantified adherence to rehabilitation exercise by employing the Rehabilitation Exercise Adherence Index (REAI), calculated as (total score/56) × 100. Higher REAI values correspond to increased adherence levels, categorized as high (>75), moderate (51–74), and low (<50).

### Tilburg frailty Indicator

The scale was developed by Gobbens et al. (24) at Tilburg University and translated into Chinese by Xi et al. (25). It comprises 15 items grouped into three dimensions: physical frailty (8 items), psychological frailty (4 items), and social frailty (3 items). The scale produces a total score between 0 and 15, with higher scores indicating increased frailty severity, and a score of ≥5 defining the frail state. Internal consistency of the scale was deemed acceptable in this study, with a Cronbach’s *α* coefficient of 0.792.

### Statistical analysis

SPSS 27.0 and R 4.4.0 were employed for the statistical analyses. Continuous data following a normal distribution are commonly depicted as (mean ± SD), whereas skewed data are represented by median (P25, P75). Group comparisons were conducted using independent sample t tests or one-way ANOVA as applicable. Multivariable analysis was performed through linear regression, resulting in the development of two regression models utilizing the resultant estimates. Model 1 remained unadjusted, whereas Model 2 underwent additional adjustment to incorporate all covariates identified as independent risk factors for the dependent variable. Restricted cubic spline (RCS) analysis was employed to fit a smooth curve and evaluate the non-linear relationship between frailty and rehabilitation exercise adherence, followed by threshold-effect detection. Sex and level of independence were ultimately incorporated to assess their interaction effects with the independent variable. The statistical significance level was established at *α* = 0.05.

### Ethical approval

This study has been approved by the Ethics Committee of the Affiliated Hospital of North Sichuan Medical College, with the ethics approval number being 2025ER49-1.

## Results

### Baseline characteristics and univariate analysis

Among the 307 participants, 179 (58.3%) were male and 128 (43.7%) were female, with a mean age of (69.41 ± 10.90) years. Living arrangements included 45 individuals (14.7%) who lived alone, whereas 262 (85.3%) resided with family members. Comorbidities were present in 262 patients (85.3%), and 80 patients (26.1%) had experienced recurrent ischemic stroke. The mean score for REAI was (54.83 ± 9.32), suggesting moderate adherence, while the mean frailty score was (4.59 ± 2.14). Univariate analysis identified gender, marital status, living arrangement, household registration type, number of medications, and level of independence as significant determinants of rehabilitation exercise adherence (Table 1).

**Table 1:** Univariate analysis of determinants affecting REAI in ischemic stroke patients.

Variables	*n* (%)	REAI	*t/F*	*P*
Gender			1.971	**0.05**
Male	179(58.31%)	55.72 ± 0.69		
Female	128(41.69%)	53.6 ± 0.82		
Age(year)			1.147	0.563
45 ~ 65	98(31.92%)	55.34 ± 7.96		
65 ~ 75	99(32.25%)	55.01 ± 11.04		
≥75	110(35.83%)	54.22 ± 8.79		
Marital status			3.504	**<0.001**
Married	230(74.92%)	55.89 ± 0.6		
Unmarried/divorced/widowed	77(25.08%)	51.67 ± 1.06		
Living arrangement			−3.619	**<0.001**
Alone	45(14.66%)	50.28 ± 9.16		
Family	262(85.34%)	55.62 ± 9.14		
Education level			−0.783	0.434
Primary school or below	201(65.47%)	54.53 ± 9.75		
Middle school or above	106(34.53%)	55.41 ± 8.45		
Household registration type			2.087	**0.038**
Urban	146(47.56%)	55.99 ± 9.27		
Rural	161(52.44%)	53.78 ± 9.26		
Insurance			−0.059	0.953
Yes	245(79.80%)	54.82 ± 9.29		
No	62(20.20%)	54.9 ± 9.53		
Occupation			1.698	0.185
On the job	38(12.38%)	56.53 ± 7.11		
Retirement	72(23.45%)	55.9 ± 9.41		
Unemployed	197(64.20%)	54.12 ± 9.62		
Current smoking status			0.839	0.402
Yes	93(30.29%)	55.51 ± 9.96		
No	214(69.71%)	54.54 ± 9.04		
Current alcohol status			0.12	0.905
Yes	85(27.69%)	54.94 ± 9.33		
No	222(72.31%)	54.79 ± 9.34		
Number of strokes			1.286	0.2
First occurrence	227(73.94%)	55.24 ± 9.26		
Recurrence	80(26.06%)	53.68 ± 9.45		
Comorbidities			−0.253	0.801
Yes	262(85.34%)	54.78 ± 9.4		
No	45(14.66%)	55.16 ± 8.95		
Number of medications			3.463	**0.033**
0 ~ 2	125(40.72%)	53.89 ± 8.86		
3 ~ 4	117(38.11%)	56.59 ± 9.75		
>4	65(21.17%)	53.49 ± 9.04		
Family history			−0.34	0.734
Yes	42(13.68%)	54.38 ± 7.91		
No	265(86.32%)	54.91 ± 9.54		
Level of independence			69.922	**<0.001**
Mostly independent	130(42.35%)	60.29 ± 9.01		
Minimally independent	118(38.44%)	53 ± 7.08		
Completely dependent	59(19.22%)	46.49 ± 5.66		

Note: Values in bold in indicate *P*≤0.05.

### Linear regression analysis of the effect of frailty on rehabilitation exercise adherence

Rehabilitation exercise adherence was used as the dependent variable, with covariates showing significance in univariate analyses included. Gender and level of independence were identified as independent risk factors for adherence through linear regression analysis. Model 1 (unadjusted) demonstrated that an increase of one point in frailty was associated with a decrease of 2.66 points in the adherence index (*p* < 0.001). In Model 2, which additionally adjusted for sex and independence level, each one-point increase in frailty was associated with a 2.06-point decrease (*p* < 0.001). In both models, frailty was consistently identified as an independent risk factor for impaired rehabilitation exercise adherence (Table 2).

**Table 2:** Threshold effect analysis of the association between frailty and the rehabilitation exercise adherence.

Linear regression						Piecewise linear regression					Model comparison		
	***β***	**95%CI**	***t***	***P***	**logLik**		***β***	**95%CI**	***t***	***P***	**logLik**	**χ** ^**2**^	***P***
Model 1					−1048.25	Model 1					−1041.48	13.53	<0.001
(Intercept)	67.05	65.09 ~ 69.01	67.28	<0.001		(Intercept)	62.61	59.03 ~ 66.19	34.41	<0.001			
Frailty	−2.66	−3.05 ~ −2.28	−13.53	<0.002		Frailty_low	−1.24	−2.28 ~ −0.2	−2.34	0.02			
						Frailty_high	−3.18	−3.71 ~ −2.66	−12.04	<0.001			
Model 2					−1019.15	Model 12					−1013.32	11.66	<0.001
(Intercept)	67.02	65.17 ~ 68.87	71.36	<0.001		(Intercept)	62.58	59.31 ~ 65.85	37.66	<0.001			
frailty	−2.06	−2.47 ~ −1.65	−9.9	<0.001		Frailty_low	−0.58	−1.57 ~ 0.41	−1.15	0.2512			
gender (reference = male)						Frailty_high	−2.56	−3.07 ~ −2.06	−9.93	<0.001			
Female	1.87	0.26 ~ 3.48	2.28	0.023		Gender (reference = male)							
Level of independence (reference = mostly independent)						Female	1.57	−0.03 ~ 3.17	1.93	0.0542			
Minimally independent	−4.68	−6.45 ~ −2.9	−5.19	<0.001		Level of independence (reference = mostly independent)							
Completely dependent	−8.97	−11.31 ~ −6.63	−7.54	<0.001		Minimally independent	−4.9	−6.65 ~ −3.15	−5.5	<0.001			
						Completely dependent	−8.97	−11.28 ~ −6.66	−7.66	<0.001			

### Smooth-curve fitting and threshold-effect analysis of frailty compared to rehabilitation exercise adherence

**Figure 1:**
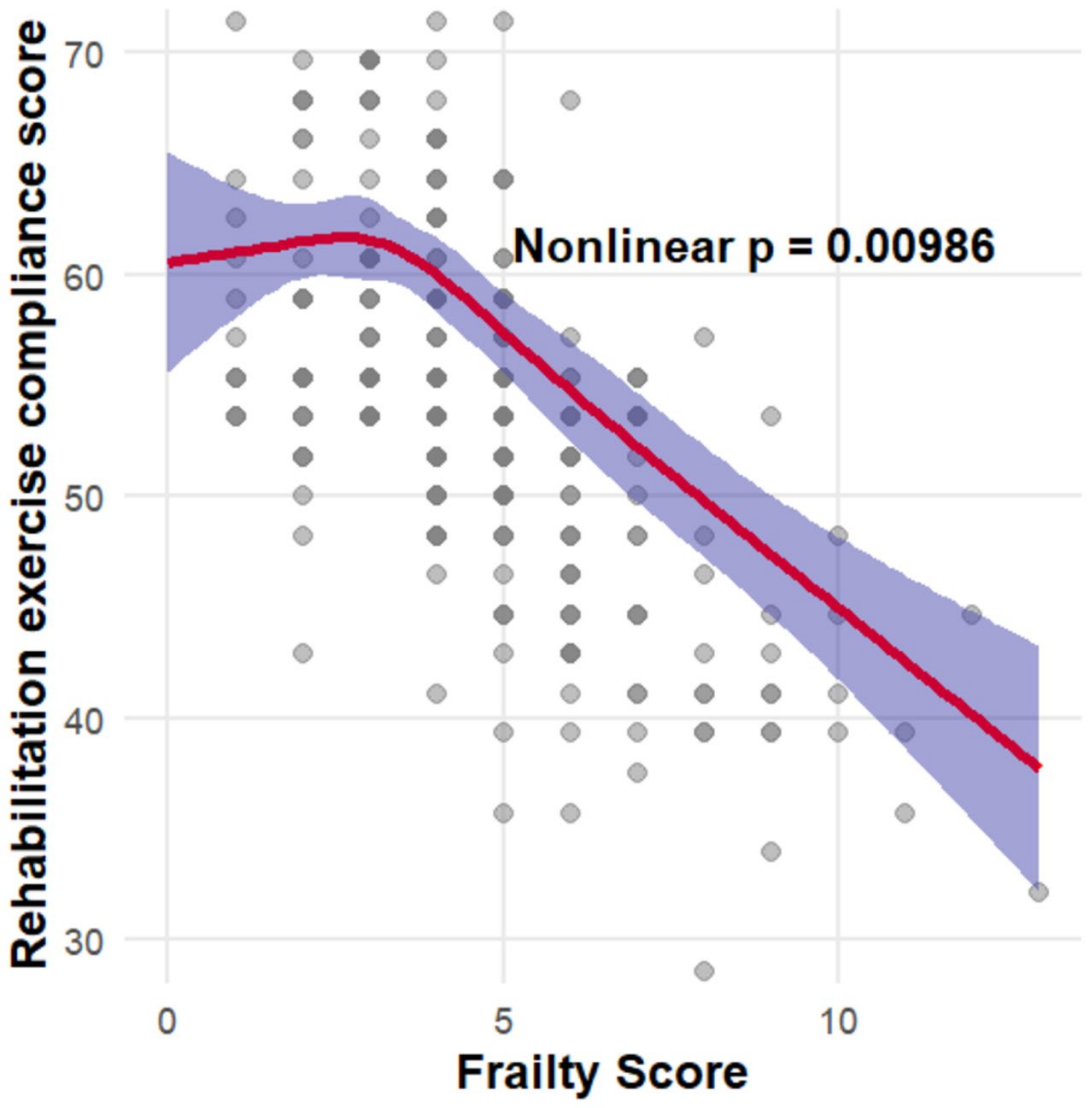
Smooth-curve fitting of frailty in relation to rehabilitation exercise adherence.

Through RCS analysis, a non-linear correlation was observed between frailty and rehabilitation exercise adherence (Non-linear *p* = 0.00986), as illustrated in Figure 1. A threshold of 3.98 (95% *CI*: 2.99–4.98) was pinpointed using a bootstrap-RCS curvature-inflection approach. Subsequently, frailty was dichotomized at this cut-off point, followed by the application of a piecewise linear regression model. In the absence of covariate adjustment, piecewise linear regression analysis demonstrated that each one-point increase in frailty was associated with a 1.24-point decline in the REAI when frailty ≤3.98 (*p* < 0.001) and a 3.18-point decline when frailty >3.98 (*p* < 0.001). After adjusting for sex and independence level, frailty had no significant effect on rehabilitation exercise adherence when scores were ≤3.98. However, above this threshold, each unit increase in frailty was associated with a 2.56-point decrease in the REAI (*p* < 0.001). Likelihood-ratio tests comparing piecewise and linear regressions, both unadjusted and adjusted, revealed higher log-likelihood values for the piecewise models, confirming a threshold effect between frailty and rehabilitation exercise adherence (Table 2).

### Interaction effect testing

**Figure 2:**
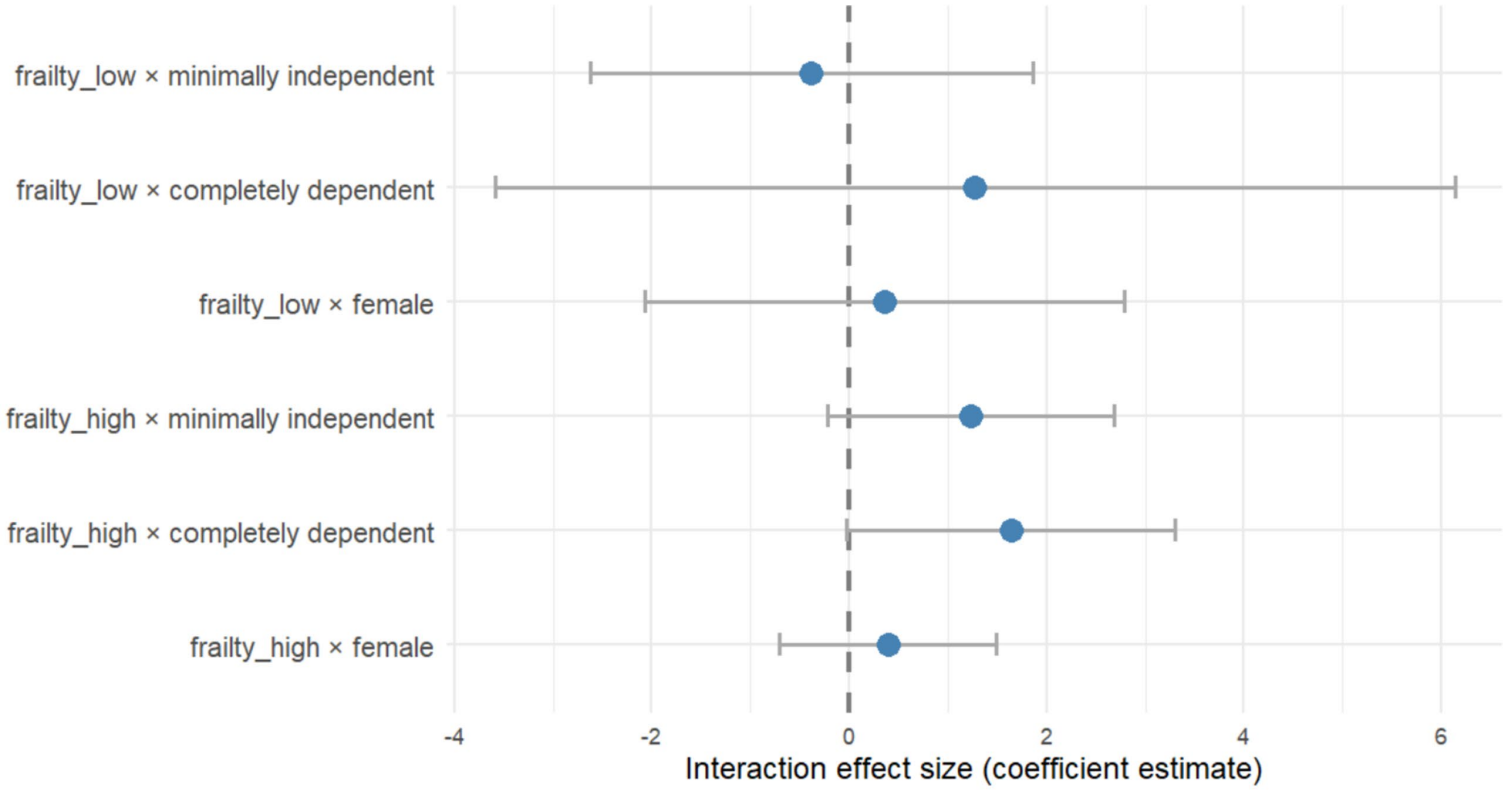
Forest plot of interaction tests between frailty and sex or independence level.

In the covariate-adjusted piecewise regression model, rehabilitation exercise adherence differed significantly across independence levels. To determine whether the threshold effect varied by level of independence, we examined the interaction between frailty and sex as well as independence. Figure 2 illustrates that none of the interaction terms were significant, indicating the robustness of the threshold effect of frailty on rehabilitation exercises adherence, and highlighting the independent yet significant impact of independence level.

## Discussion

The primary aim of this study was to determine the level of rehabilitation exercise adherence and its influencing factors among hospitalized patients with ischemic stroke, and to examine the impact of post-stroke frailty on adherence, by administering relevant self-reported questionnaires. The questionnaire classified the potential determinants of rehabilitation exercise adherence into two broad domains: (1) sociodemographic characteristics and (2) clinical features, including the number of medications and level of independence. Participants exhibited a moderate level of rehabilitation exercise adherence that remains suboptimal, with a frailty risk prevalence of 44.95%. Frailty emerged as an independent predictor of poor adherence, and a clear threshold effect was observed between frailty and rehabilitation exercise adherence.

### Determinants of rehabilitation exercise adherence

In the univariate analysis, it was determined that sex, marital status, living arrangement, household registration type, number of medications, and level of independence were all significant predictors of rehabilitation exercise adherence. Corresponding to earlier studies (26), differences in adherence between genders may be attributed to women’s higher likelihood of negative illness perception, heightened fear related to the disease, and subsequent negative emotions, all of which augment psychological burden and ultimately reduce rehabilitation exercise adherence (27). Solitary living resulting from widowhood often results in a deprivation of familial care and social support, thereby compromising adherence to rehabilitation exercises during the extended post-stroke prevention and rehabilitation period, as a consequence of the absence of daily supervision and emotional reinforcement (14, 28). Within the context of multiple chronic conditions, a higher number of medications amplifies pharmacological burden, resulting in hemodynamic fluctuations and adverse effects such as drug-related myalgia that deplete functional reserve and hinder exercise motivation (29). Urban registered patients demonstrated higher rehabilitation exercise adherence than their rural counterparts, a disparity likely attributable to unequal distribution of medical resources and divergent illness outcome perceptions between urban and rural residents (30). Independence level is a critical determinant of post-stroke rehabilitation exercise adherence; it is dually modulated by the extent of motor impairment and psychological status (27). Functional impairments directly impede the ability to engage in exercises, while psychological distress weakens behavioral drive, collectively fostering a self-perpetuating cycle that accelerates the decline in adherence.

### Current status of rehabilitation exercise adherence and frailty in patients with ischemic stroke

The mean REAI was 54.83 ± 9.32, indicating moderate adherence and aligning with previous findings (31). Rehabilitation exercise adherence is strongly predictive of post-stroke outcomes. Research suggests that while adherence is initially high, it tends to decrease as the rehabilitation period progresses, resulting in an overall downward trajectory over time (32–34). Post-stroke somatic symptoms increase discomfort and risk sensitivity in older, multimorbid, and severely debilitated patients, leading to skepticism toward rehabilitation exercises and reducing adherence. Additionally, the prolonged recovery process, often without adequate professional supervision and coordination, contributes to a significant decrease in adherence. Moreover, the observed frailty prevalence of 44.95% in this study aligns with previously reported rates ranging from 28 to 52.5% (18, 35, 36). The higher prevalence observed in our study could be attributed to severe functional impairment, prolonged hospitalization, and multimorbidity. Frailty, characterized by diminished multi-organ reserve, is evident through slowed gait, reduced endurance, and heightened fatigue, which directly compromise motor capacity and hinder functional recovery and clinical outcomes (37).

### Impact of frailty on rehabilitation exercise adherence and its threshold effect

The results of our study indicate that frailty independently contributes to suboptimal adherence to prescribed rehabilitation exercise. Consistent with previous research (38), pulmonary rehabilitation can effectively mitigate frailty in patients diagnosed with chronic obstructive pulmonary disease. However, the failure to maintain sufficient adherence significantly heightens the risks of both frailty progression and accelerated deterioration in pulmonary function. These data indicate that frailty-related symptoms exert a deleterious effect on rehabilitation adherence, whereas sustained engagement in exercise training not only reverses frailty but also yields meaningful disease-specific improvements. Frailty exerts a detrimental impact on adherence to therapeutic recommendations in hypertensive cohorts. Health literacy is identified as a key mediator within this pathway, with educational attainment serving as an effect modifier (39, 40). Frailty also hinders compliance with prescribed exercise rehabilitation among cancer patients (41). Emerging evidence indicates that personalized exercise regimens tailored to individual needs could help reduce the negative effects of frailty on adherence to rehabilitation programs (13). Collectively, these findings indicate that frailty is a pivotal, multidimensional determinant of rehabilitation and therapeutic adherence across diverse disease contexts. When prescribing exercise interventions for stroke survivors, clinicians should therefore extend their focus beyond the neurological insult and sociodemographic characteristics to systematically assess and actively manage frailty; mitigating frailty itself constitutes a critical leverage point for enhancing adherence.

Through smooth-curve fitting and threshold analysis, a curvilinear correlation between frailty and rehabilitation exercise adherence was revealed, displaying an inflection point at 3.98. Adherence levels exhibited stability for frailty scores ≤3.98, but demonstrated a marked decrease beyond this threshold: every additional point in frailty past the threshold was associated with a reduction of 2.56 points in the adherence index (*p* < 0.001). The impact of frailty on rehabilitation exercise adherence likely arises from the interplay of multiple, mutually reinforcing factors. Neurological impairment from stroke precipitates physical frailty, manifesting as diminished endurance and capacity, which directly constrains exercise ability and fosters avoidance behavior. Simultaneously, psychological frailty, influenced by post-stroke anxiety, depression, and limited health literacy, diminishes motivation and expectations of recovery. Diminished social roles and inadequate social support further attenuate the impetus to sustain rehabilitation exercise (42–44). Importantly, declining adherence further exacerbates frailty, establishing a “frailty–low adherence–greater frailty” vicious cycle that can be interrupted by early intervention to improve outcomes. Frailty is defined as ≥5 points by the TFI; the observed inflection at 3.98 probably signifies pre-frailty, a transitional phase where key frailty symptoms emerge and which is also associated with notable clinical harms (24). Compared with non-frail individuals, pre-frail populations exhibit a 2- to 3-fold higher risk of increased disease susceptibility, impaired balance, and falls (45). Additionally, approximately 10–15% of pre-frail individuals progress to overt frailty annually (46). At this stage, patients may already exhibit diminished capacity and motivation, leading to progressively lower rehabilitation exercise adherence. Additionally, adherence to rehabilitation exercises, a standard behavioral parameter, is inherently sensitive to initial, more nuanced functional changes. Even before frailty criteria are met, pre-frail individuals may experience mild fatigue or psychological reluctance that reduces exercise frequency and lowers adherence (47). Moreover, our study population of ischemic stroke patients demonstrates diminished physiological reserves and heightened susceptibility to frailty. The compounding effects of stroke-induced disability and underlying comorbidities magnify the early impact of frailty on exercise adherence (48, 49). Owing to the cross-sectional design, we cannot establish the temporal sequence or infer causality between frailty and rehabilitation exercise adherence. Prospective longitudinal or intervention studies are therefore warranted to elucidate this causality. Importantly, our findings consistently demonstrate that frailty independently and substantially influences adherence, providing a robust foundation for future mechanistic research and targeted clinical interventions.

## Strengths and limitations

A frailty score >3.98 was identified as a critical intervention cut-off through threshold-effect analysis. Addressing frailty at this threshold has the potential to prevent its progression and notably enhance rehabilitation exercise adherence in ischemic stroke patients. Several limitations should be acknowledged. Firstly, the convenience sample was derived from only two hospitals, thus constraining generalizability and potentially introducing selection bias. Secondly, the cross-sectional nature of the study precludes making causal inferences; therefore, there is a need for multi-center, large-scale longitudinal investigations. Lastly, frailty assessment was conducted 1 day before discharge; including pre-stroke frailty metrics would help elucidate the specific impacts of pre-existing frailty compared to post-stroke frailty on adherence to rehabilitation.

## Conclusion

Rehabilitation exercise adherence among ischemic stroke patients in this study was moderate and remains to be improved. Additionally, frailty is highly prevalent among stroke survivors and constitutes an independent risk factor for reduced rehabilitation exercise adherence. Threshold analysis revealed that rehabilitation exercise adherence declines sharply once frailty scores exceed 3.98. This finding indicates that the pre-frail stage represents a critical intervention window; personalized and diversified adjustments to rehabilitation programs initiated during this phase are likely to be more effective than interventions delivered after overt frailty has developed, thereby averting a self-perpetuating “frailty–low adherence” cycle.

## Data Availability

The raw data supporting the conclusions of this article will be made available by the authors, without undue reservation.
